# Measuring kindergarteners’ motivational beliefs about writing: a mixed-methods exploration of alternate assessment formats

**DOI:** 10.3389/fpsyg.2023.1217085

**Published:** 2023-08-04

**Authors:** Megumi E. Takada, Christopher J. Lemons, Lakshmi Balasubramanian, Bonnie T. Hallman, Stephanie Al Otaiba, Cynthia S. Puranik

**Affiliations:** ^1^Graduate School of Education, Stanford University, Stanford, CA, United States; ^2^Simmons School of Education and Human Development, Southern Methodist University, Dallas, TX, United States; ^3^College of Education and Human Development, Georgia State University, Atlanta, GA, United States

**Keywords:** motivation, writing, kindergarten, assessment, literacy, interview, survey, mixed methods

## Abstract

There have been a handful of studies on kindergarteners’ motivational beliefs about writing, yet measuring these beliefs in young children continues to pose a set of challenges. The purpose of this exploratory, mixed-methods study was to examine how kindergarteners understand and respond to different assessment formats designed to capture their motivational beliefs about writing. Across two studies, we administered four assessment formats — a 4-point Likert-type scale survey, a binary choice survey, a challenge preference task, and a semi-structured interview — to a sample of 114 kindergarteners engaged in a larger writing intervention study. Our overall goals were to examine the benefits and challenges of using these assessment formats to capture kindergarteners’ motivational beliefs and to gain insight on future directions for studying these beliefs in this young age group. Many participants had a difficult time responding to the 4-point Likert-type scale survey, due to challenges with the response format and the way the items were worded. However, more simplified assessment formats, including the binary choice survey and challenge preference task, may not have fully captured the nuances and complexities of participants’ motivational beliefs. The semi-structured interview leveraged participants’ voices and highlighted details that were overlooked in the other assessment formats. Participants’ interview responses were deeply intertwined with their local, everyday experiences and pushed back on common assumptions of what constitutes negatively oriented motivational beliefs about writing. Overall, our results suggest that kindergarteners’ motivational beliefs appear to be multifaceted, contextually grounded, and hard to quantify. Additional research is needed to further understand how motivational beliefs are shaped during kindergarten. We argue that motivational beliefs must be studied in context rather than in a vacuum, in order to work toward a fair and meaningful understanding of motivational beliefs about writing that can be applied to school settings.

## 1. Introduction

Developing the skill to put thoughts into words and then transcribe these words so that another person can understand the thoughts is one of the principal learning objectives in most educational settings across the globe. Writing systems are the foundation of literacy, and humans have been engaged with written communication as far back as 35,000 BCE (Fischer, 2021). Over time, writing has evolved into a complex social activity situated within sociocultural contexts. As young children today encounter writing in their environment, and especially as they enter school, they learn to engage in writing to express themselves and communicate within these broader communities.

Over the years, scholars from various disciplines have studied writing from both cognitive perspectives (e.g., Hayes and Flower, 1980; Bereiter and Scardamalia, 1987) and sociocultural perspectives (e.g., Barton and Hamilton, 1998). Such theoretical frameworks have led to a rich understanding of writing as both a complex, mental process that requires orchestration of a wide range of cognitive skills, as well as a social process that occurs between individuals (Graham, 2018; Rowe, 2023). More recently, Graham (2018) proposed the writer(s)-within-community model that combines earlier cognitive and sociocultural perspectives to extend our understanding of the dynamic interactions between the characteristics of the writer, of the writing community, and the written product.

Among the many intricate processes involved in writing are motivational beliefs about writing. Studies examining motivational beliefs about writing have gained traction in the past few decades, as some of the earliest cognitive models of writing (e.g., Hayes and Flower, 1980) were revised to include such affective factors (e.g., Hayes, 1996). Stemming from a wide range of theories, motivational beliefs about writing are multidimensional. A variety of motivation-related constructs have been studied; however, many of these studies lack clear operational definitions of the constructs being examined (cf. Camacho et al., 2021). Further, authors also seem to use various terms interchangeably. Here, we adopt the term *motivational beliefs* from Graham (2022) in its plural form to cast light on the various aspects of the construct. Graham (2018, pp. 266–267) describes seven sets of motivational beliefs about writing: (1) “judgments about the value and utility of writing or expectancy-value beliefs,” (2) “beliefs involv[ing] whether one likes to write … or views writing as an attractive activity,” (3) “views about writing competence,” (4) “beliefs focus[ing] on why one engages in writing,” (5) “judgements about why one is or is not successful,” (6) “beliefs about their identities as writers,” and (7) “beliefs about writing communities.” For the remainder of the manuscript, we will use *motivational beliefs* to refer to motivational beliefs about writing.

The increasing amount of attention on motivational beliefs following Hayes’s (1996) work has gone beyond just the research community. Following Dweck’s (2006) best-selling book on growth mindset — the belief that abilities can change through persistent work — there has been an increasing popularity among education practitioners and parents around fostering motivation in general. Despite this widespread, public interest, researchers have not come to a clear understanding of the role of motivational beliefs in writing. In a systematic review of research published between 2000 and 2018, Camacho et al. (2021) found that overall, research showed a weak-to-moderate, positive relation between motivational beliefs and writing performance. However, their synthesis only included participants in 1st-12th grade. Other populations, such as kindergarteners, were excluded. Overall, it is unclear whether motivational beliefs facilitate writing growth, and the outcomes from studies examining relations between motivational beliefs and writing skills have been highly variable (Graham, 2022). The increased public attention on motivational beliefs, combined with the limited empirical knowledge available to guide decision making, underscores the critical need for additional research in this area.

### 1.1. Kindergarteners’ motivational beliefs about writing

Graham (2022) highlighted the importance of studying motivational beliefs in a wider age range. Given that research examining kindergarteners’ motivational beliefs about writing is limited, there is a dire need to extend this research to this younger age group. A better understanding of kindergarteners’ motivational beliefs is critical to support young writers in their early school years. In kindergarten, most children are exposed to their first year of formal writing instruction. During this time when children are forming their early identities as writers, it is important that we provide environments that establish and maintain positive motivational beliefs. From a developmental standpoint, examining the early stages of such motivational beliefs is likely to enrich our understanding of the ways in which these beliefs dynamically change across grade levels.

Among the few studies that have analyzed kindergarteners’ motivational beliefs, researchers have approached motivational beliefs from different perspectives and have asked a variety of research questions. Nolen (2001) conducted an ethnographic study, documenting the ways in which local, sociocultural contexts (e.g., classroom literacy practices, teacher beliefs, and student-to-student interactions) shaped kindergarteners’ motivation to read and write. Kim and Lorsbach (2005) focused specifically on writing self-efficacy — the belief that one can successfully complete a task — and examined whether children as young as kindergarteners can express their own self-efficacy. Even though language and cognitive skills are still developing at this age, they found that kindergarteners were able to express their own self-efficacy using words, attitudes, and behaviors. Others have aimed to characterize motivational beliefs and found that kindergarteners generally have positive beliefs about writing; many of them sustain an interest in writing throughout the school year (Nolen, 2001) and are highly motivated to write (Mata, 2011). Finally, in a more recent exploratory study, Schrodt et al. (2019) found that combining instruction on writing and mindset/self-regulation increased kindergarteners’ writing motivation. Altogether, these studies span a wide range of topics, but additional research is needed to establish a more robust research foundation.

### 1.2. Measuring kindergarteners’ motivational beliefs

A commonly reported challenge in studies with young children is the difficulty of measuring motivational beliefs in this age group (e.g., Turner, 1995; Kim and Lorsbach, 2005; Schrodt et al., 2019, 2022). In fact, this challenge is likely to be one of the main reasons why there is such a limited number of studies with kindergarteners. In order to study motivational beliefs in kindergarteners, more research is needed to understand *how* to capture their motivational beliefs in the first place. Taking the time to carefully explore this question is critical to running any study on kindergarteners’ motivational beliefs. It is only after we gain a fuller understanding of what we are measuring that we can be more confident in the interpretations we make from the results.

Many challenges stand in the way of accurately capturing these beliefs in young children. Given that internal thought processes such as motivational beliefs are difficult to observe, self-report measures are often used. However, in using such self-report measures, young children may not be developmentally ready to fully reflect and explain their beliefs accurately from both a cognitive and linguistic standpoint (Kim and Lorsbach, 2005). Indeed, young children find it challenging to answer generalized statements commonly used in surveys, as they tend to think more concretely about specific situations, oftentimes ones that they just experienced (Turner, 1995). Additionally, young children frequently provide answers that represent the extremes of Likert-type questions (Mellor and Moore, 2014; Ruzek et al., 2020), self-report in an overwhelmingly positive manner (Gambrell and Gillis, 2007), and are generally more optimistic (Turner, 1995), resulting in overinflated accounts of motivation. While such accounts of motivation may be a reflection of the limited amount of negative academic experiences that children at this young age have (Gambrell and Gillis, 2007), tendencies to positively self-report may also be due to social desirability bias. In fact, self-reports do not always align with student behavior and performance (Turner, 1995; Graham et al., 2017). These issues make self-report measures, such as surveys and interviews, challenging to use.

While many of these challenges cannot be easily addressed, past research has acknowledged some of these challenges and have taken steps to make self-report measures more developmentally appropriate. For example, Nolen’s (2001) student interview measure, Mata’s (2011) Motivation for Reading and Writing Profile survey, and Schrodt et al.’s (2019) Literacy and Writing Motivation Survey all used a response format aimed at reducing social desirability bias. In these measures, participants were introduced to two stuffed animals with different motivational belief profiles, then asked to choose the one they are more like. This format legitimized both choices through a more neutral presentation of the two profiles (Baker and Scher, 2002). Efforts have also been made to simplify wording, such as by adapting the wording of items designed for upper elementary school children to meet the needs of a younger age group (Kim and Lorsbach, 2005). Other related surveys measuring young children’s reading motivation have additionally used visual aids (e.g., happy/sad faces), consistent response formats across all items, and items that reflect specific, concrete scenarios that young children can more easily relate to (Baker and Scher, 2002; Wilson and Trainin, 2007).

Researchers have also leveraged qualitative and mixed-methods approaches to examine motivational beliefs. Given difficulties with using quantitative survey measures to gain an understanding of self-efficacy, Kim and Lorsbach (2005) conducted interviews and classroom observations involving kindergarteners. Similar ethnographic methods were also used by Nolen (2001) who used a hybrid approach in which participants completed a Likert-type scale survey, while interviewers recorded participants’ commentary as they engaged with the survey. Noting challenges in accurately measuring kindergarteners’ motivational beliefs, Schrodt et al. (2019) aimed to triangulate evidence through mixed methods (i.e., a survey, an interview, and a behavioral task).

Recent efforts have also focused specifically on expanding upon typical self-report measures by assessing motivational beliefs through a behavioral task. Schrodt et al. (2022) conducted further analyses on a behavioral task, the Writing Challenge Task, used in their earlier work (Schrodt et al., 2019). This behavioral, task-based assessment measures challenge preferences during writing as a means to capture motivational beliefs. The authors found that scores on the Writing Challenge Task predicted kindergarteners’ end-of-year writing performance. While capturing kindergarteners’ motivational beliefs continues to pose challenges, such studies contribute to the field’s efforts to reflect and further improve on ways to study young children’s motivational beliefs.

### 1.3. The present studies

Across two studies, we aimed to address the challenges involved in measuring kindergarteners’ motivational beliefs about writing by examining four different assessment formats: a 4-point Likert-type scale survey, a binary choice survey, a challenge preference task, and a semi-structured interview. Specifically, we asked the following research questions: (1) Do kindergarteners understand these assessment formats? (2) How do they respond to these assessment formats? and (3) Are motivational beliefs about writing (as measured by the 4-point Likert-type scale survey, binary choice survey, and challenge preference task) related to writing skills?

Our overall goals were to examine the benefits and challenges of using these assessment formats to capture kindergarteners’ motivational beliefs and to gain insight on future directions for studying these beliefs in this young age group. Importantly, these studies did not aim to formally validate included assessments. Rather, our studies were exploratory, leveraging the opportunity to compare several different assessment formats to provide a unique, more comprehensive lens in which to address the aims of our research. In both studies, we use the term *motivational beliefs* to specifically refer to “views about writing competence” (p. 266) and “judgements about why one is or is not successful” (p. 267) (Graham, 2018).

Both studies were embedded within a larger project conducted during the 2021–22 school year. The goal of the larger project was to examine the initial efficacy of peer-assisted writing strategies (PAWS; Puranik et al., 2018), a fully developed education intervention to teach transcription and sentence generation to kindergarteners. Due to interest in measuring motivational beliefs, we piloted a measure during the pre-intervention assessment period in the first month of the school year (i.e., Study 1). Specifically, we examined a 4-point Likert-type scale survey, which we adapted from a measure that was used in our prior research (Al Otaiba et al., 2020; Tock et al., 2021). The 4-point Likert-type scale survey was difficult for many participants to complete (e.g., participants agreed with all items regardless of whether the items were negatively or positively oriented). Based on this overall finding from Study 1, we conducted Study 2 examining three additional assessment formats: a binary choice survey, a challenge preference task, and a semi-structured interview. Our rationale for this second study was that these alternate assessment formats could be better suited to the needs of young children. Specifically, we aimed to make the assessment formats more developmentally appropriate for kindergarteners. For example, we made tasks simpler (e.g., by reducing the number of survey response choices), more straightforward (e.g., by having participants complete a more concrete, task-based, behavioral assessment), and more flexible (e.g., by asking open-ended interview questions). Study 2 occurred toward the end of the school year during the post-intervention assessment period, about seven months after Study 1. Although our focus in Studies 1 and 2 was not related to the effect of the writing intervention, we did conduct initial analyses for Study 2 to ensure that our variables of interest did not differ by condition (i.e., treatment/control). We did not find any group differences and thus combined the two groups for all further analyses.

One hundred and fourteen kindergarten children (mean age: 5.46 years old, range: 4.92–6.08 years old; female: *n* = 58) from six classrooms in three public school districts in Northern California participated in both Studies 1 and 2. According to school records, 32% of participants were White, 27% were Hispanic/Latinx, 19% were multi-racial, 17% were Asian, 1% were Native Hawaiian/other Pacific Islander, 1% were classified as “other,” 0% were American Indian/Alaska Native, and 0% were Black/African American. Percentages sum to less than 100%, as information was not available for four participants. Twenty-one percent of participants were eligible for free and reduced-price meals. In Study 1, data from 110 of the 114 participants were analyzed, after accounting for absences (*n* = 3) and unusable data due to tester error (*n* = 1). In Study 2, data were analyzed from 104 of the 114 participants, after accounting for absences (*n* = 1), participants who had transferred to another school or class since Study 1 (*n* = 2), and unusable data due to tester error (*n* = 7). See Table 1 for more information on the demographics of the participants.

**Table 1:** Demographics of participants (*N* = 114).

Demographic	*n*	Mean	*SD*
Age		5.46	0.31
Sex assigned at birth			
Female	58		
Male	56		
Race/ethnicity			
White	36		
Hispanic/Latinx	31		
Multi-racial	22		
Asian	19		
Native Hawaiian/other Pacific Islander	1		
American Indian/Alaska Native	0		
Black/African American	0		
Classified as “other”	1		
Data not available	4		
Free/reduced price meals			
Eligible	24		
Not eligible	89		
Data not available	1		

*SD* represents standard deviation. Age represents age in years at beginning of the school year.

## 2. Study 1

### 2.1. Materials and methods

#### 2.1.1. Measures

##### 2.1.1.1. Assessment of motivational beliefs about writing

###### 2.1.1.1.1. Four-point Likert-type scale survey

To assess motivational beliefs about writing, we administered a 4-point Likert-type scale survey, which was adapted from the Reading Mindset Measure (Al Otaiba et al., 2020; Tock et al., 2021). The Reading Mindset Measure was originally developed for upper elementary students and focused on reading. Adaptations were made to both the Likert-type scale and items.

First, we simplified the original 6-point Likert-type scale into a 4-point Likert-type scale (1 = *Definitely disagree*, 2 = *Kind of disagree*, 3 = *Kind of agree*, 4 = *Definitely agree*). Likert-type scales for younger age groups are often simplified, ranging from 3-points to 5-points (Mellor and Moore, 2014). Following guidance that midpoints (e.g., 3-point or 5-point) should only be used when respondents are familiar with the topic (Chyung et al., 2017), we decided to use a 4-point scale. We further provided visual scaffolding (Reynolds-Keefer and Johnson, 2011) by accompanying the Likert-type scale with a visual of a thumb facing downwards to upwards. While past research has used visuals of happy/sad faces with young children (Wilson and Trainin, 2007), we felt that thumb signals would be more emotionally neutral compared to faces and more representative of degrees of dis/agreement. Thumb signals also provided participants with a non-verbal mode of communication, which we believed would encourage more honest responses and ease tension that some participants may experience in answering questions that felt personal.

Items were reworded to reflect motivational beliefs about writing. For example, the item “If a book is hard to read, I stop reading it.” (Al Otaiba et al., 2020; Tock et al., 2021) was changed to “If a word is hard to write, I stop writing it.” All seven items in the Reading Mindset Measure were reworded in this manner (see Supplementary Table 1). All items in the original Reading Mindset Measure assessed a negative orientation to motivational beliefs. As elementary-aged children and especially younger children are known to have difficulty disagreeing with negatively oriented items (Benson and Hocevar, 1985; Marsh, 1986), we additionally added three items that assessed a positive orientation to motivational beliefs (e.g., “I think I can keep getting better at writing words.”) (see Supplementary Table 1).

**Figure 1:**
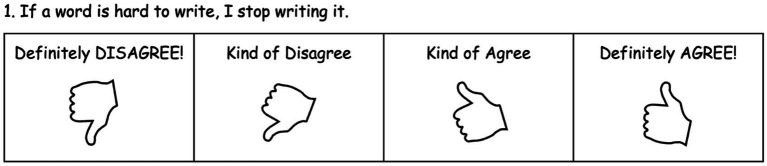
Sample item from a 10-item, 4-point Likert-type scale survey designed to measure kindergarteners’ motivational beliefs about writing. The survey included items that measured negative orientations to motivational beliefs (*n* = 7) and positive orientations to motivational beliefs (*n* = 3). Testers worked individually with participants, reading out items to participants as participants circled their responses.

In total, the 4-point Likert-type scale survey included 10 randomly ordered items. Trained testers read out each item, and participants circled their responses. At the end, the testers rated participants’ level of understanding (1 = Did not understand the activity, 2 = May not have understood the activity, 3 = Clearly understood the activity). Composite scores were computed by reverse scoring negatively oriented items, then summing all 10 items (possible range: 10–40). Lower scores were intended to reflect a more negative orientation to motivational beliefs, and higher scores were intended to reflect a more positive orientation. See Figure 1 for a sample item and Supplementary Appendix A for the full measure with administration procedures.

##### 2.1.1.2. Assessment of writing

The Wide Range Achievement Test, Fifth Edition (WRAT-5) Spelling Subtest, Blue Response Booklet (Wilkinson and Robertson, 2017) was used to measure skills in writing letters and words. This standardized assessment has two parts: Letter Writing and Spelling. In Letter Writing, participants wrote their name, as well as specific letters. In Spelling, participants spelled words (e.g., “on,” “make”) that increased in difficulty. Writing skill was operationalized as the total number of correctly answered items.

#### 2.1.2. Study procedures

A team of trained testers assessed participants one at a time during the school day. These testing sessions occurred in-person during the 2021–22 school year, as schools returned to in-person instruction after the peak of the COVID-19 pandemic. After data collection, assessment data were scored and entered, then analyzed using R Statistical Software (v4.2.1; R Core Team, 2022).

In order to examine the overall level of understanding on the survey task, we examined tester-reported ratings of participants’ understanding. Specifically, we examined the proportion of participants who (1) did not understand the task, (2) may not have understood the task, and (3) understood the task. We used Cronbach’s alpha as a measure of internal consistency, which we computed using the *psych* package (Revelle, 2022).

To understand how participants responded to the survey, we compared response patterns of participants who understood the task and those who did not understand the task. Specifically, we examined how they responded to positively and negatively oriented items. We ran a two-way ANOVA predicting response patterns by level of understanding (did not understand task/understood task) and item type (negatively/positively oriented) (e.g., in R: proportion of definitely agree ~1 + understanding + item type + understanding:item type). We used the *joint_tests()* function in the *emmeans* package (Lenth, 2022) to extract the results of the two main effects and the interaction effect. We further examined the skewness of response distributions using the *sur* package (Harel, 2020).

The relation between motivational beliefs and writing skills was examined by fitting a linear regression model predicting writing skills using the composite score of motivational beliefs (in R: writing skills ~1 + motivational beliefs). We did not add age as a control variable, given that all participants were in the same grade. Throughout data analysis, the *tidyverse* package (Wickham et al., 2019) was used for data wrangling and data visualization.

### 2.2. Results

#### 2.2.1. Four-point Likert-type scale survey

Participants appeared to have varying levels of understanding on the survey task. Tester ratings were as follows: 35% of participants (*n* = 38) clearly understood the task, 33% (*n* = 36) may not have understood the task, and 32% (*n* = 35) did not understand the task. Tester ratings were not available for one participant, due to tester error. Internal consistency among the 10 items was moderate (*α* = 0.66).

**Figure 2:**
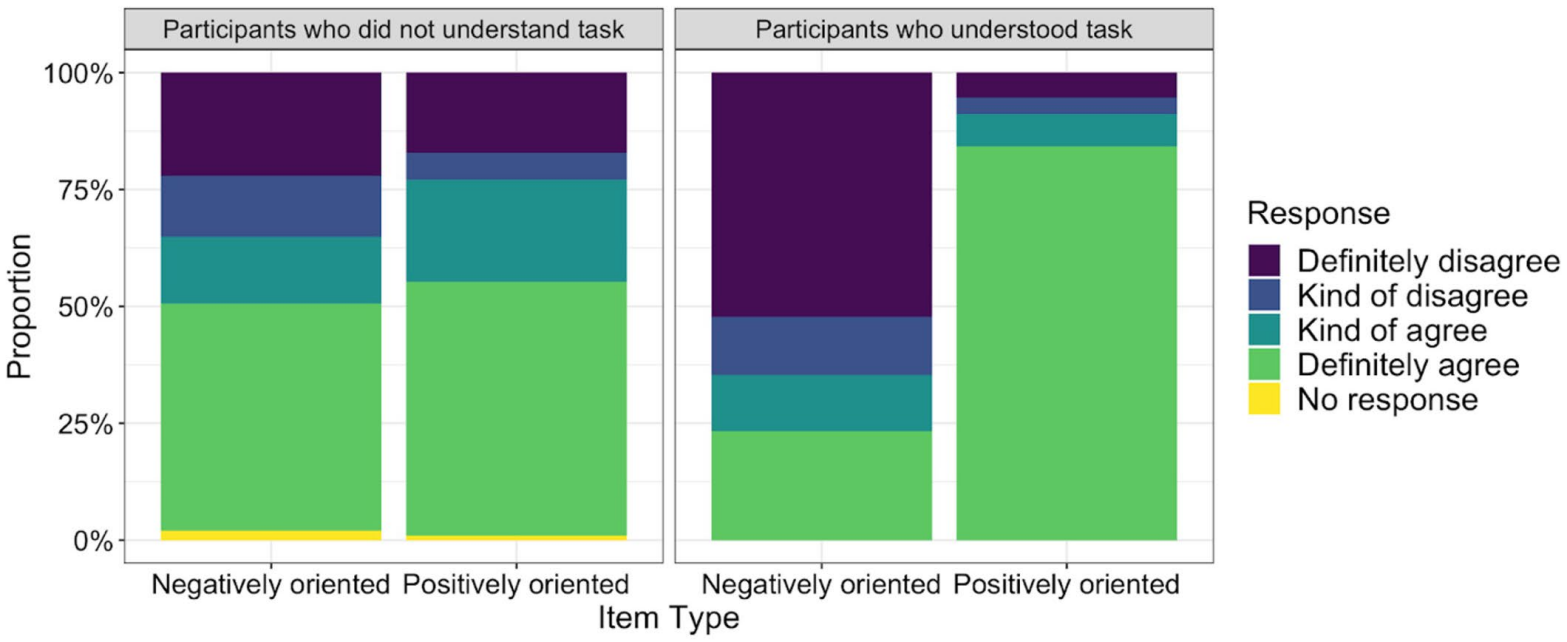
Response patterns of the 10-item, 4-point Likert-type scale survey by item type (negatively/positively oriented) and participants’ level of understanding (did not understand task/understood task). Participants completed a 4-point Likert-type scale survey designed to measure motivational beliefs about writing. Testers rated how well participants understood the task (*n* = 35 did not understand task, *n* = 38 understood task). The survey included seven negatively oriented items and three positively oriented items. Within each item type and level of understanding, we calculated the proportion of times participants chose each response category (i.e., *Definitely disagree, Kind of disagree, Kind of agree, Definitely agree, and No response*).

In order to analyze how the participants responded to the 4-point Likert-type scale survey, we examined response patterns of the survey items. Given that at least a third of participants had difficulty understanding the task, we specifically looked at the distribution of responses by level of understanding. For ease of interpretation, we focused on the groups of participants who had the highest and lowest levels of understanding (i.e., those who clearly understood the task and those who did not understand the task). Figure 2 shows the response patterns of both negatively and positively oriented items in these two groups. For those who did not understand the task, the most popular response was *definitely agree* for both negatively oriented items (*n* = 119, 49%) and positively oriented items (*n* = 57, 54%). In contrast, participants who understood the task had a tendency to definitely disagree with negatively oriented items (*n* = 139, 52%) and definitely agree with positively oriented items (*n* = 96, 84%). In other words, the most popular response for negatively oriented items differed by group (i.e., *definitely agree* for those who did not understand the task and *definitely disagree* for those who understood the task); however, response patterns for positively oriented items were more similar, as both groups were most likely to definitely agree. Response patterns for each of the 10 survey items are included in Supplementary Figure 1.

These observations were in line with results from a two-way ANOVA. We predicted participants’ response patterns by level of understanding (did not understand task/understood task) and item type (negatively/positively oriented). In a model predicting the proportion of times participants strongly agreed, there was no main effect of level of understanding, *F*(1, 123) = 0.75, *p* = 0.390, a main effect of item type, *F*(1, 123) = 64.33, *p* < 0.001, and an interaction effect between the two variables, *F*(1, 123) = 25.77, *p* < 0.001. For the proportion of times participants strongly disagreed, we found no main effect of level of understanding, *F*(1, 67) = 0.05, *p* = 0.821, no main effect of item type, *F*(1, 67) = 0.17, *p* = 0.678, and an interaction effect between the two variables, *F*(1, 67) = 5.93, *p* = 0.018.

Participants who clearly understood the task had a strong tendency to self-report positive orientations to motivational beliefs. After reverse scoring negatively oriented items, we examined the distribution of responses across all items and found that the distribution was skewed (skewness = −0.97, *SE* = 0.13). This skew was driven by participants’ responses to positively oriented items. A highly skewed distribution was observed across positively oriented items (skewness = −2.64, *SE* = 0.23). Negatively oriented items led to more varied responses, resulting in a less skewed distribution (skewness = 0.59, *SE* = 0.15).

We further explored whether motivational beliefs were related to writing skills. Given tester ratings, we were not confident that all participants’ survey data were valid. We therefore only included participants who appeared to clearly understand the task (*n* = 38). We found that motivational beliefs were not significantly related to writing skills, *F*(1, 36) = 3.54, *p* = 0.068, *r* = 0.30.

### 2.3. Study 1 summary

In Study 1, we examined the use of a 4-point Likert-type scale survey to measure kindergarteners’ motivational beliefs about writing. Despite the adaptations we made to the original survey designed for older children, the survey was difficult for many participants to complete. There was a noticeable trend among at least a third of the participants to agree with all items, regardless of whether the items were negatively or positively oriented. Survey responses, especially those for negatively oriented items, may have been affected by acquiescence bias. Responding to negatively oriented items with varying degrees of dis/agreement is likely to have been too cognitively taxing for many of the participants. While these findings may suggest that positively oriented items are generally a better measure of kindergarteners’ motivational beliefs, positively oriented items also led to highly skewed response distributions. Compared to positively oriented items, negatively oriented items may have provided a more sensitive measure of motivational beliefs in participants who clearly understood the task. Across all items, many participants also reported in ways that reflected positive orientations to motivational beliefs, possibly due to social desirability bias. Motivational beliefs were not related to writing skills. Altogether, these results and implications prompted the use of another set of assessment formats that set the stage for Study 2.

## 3. Study 2

### 3.1. Motivation for Study 2

In Study 2, we tested three additional assessment formats of motivational beliefs about writing. We aimed to make these assessment formats more developmentally appropriate by making tasks simpler, more straightforward, and more flexible. These assessment formats were designed to address the challenges that surfaced in Study 1 and to explore methods that could enhance the validity of responses. First, we examined a binary choice survey to explore the utility of a survey with a simpler response format that was more neutral and less cognitively taxing. Second, we examined a challenge preference task to explore the possibility of assessing motivational beliefs through a more concrete, task-based, behavioral assessment. Third, we took a step back, using a more open-ended, semi-structured interview to leverage the voices of kindergarteners and observe the ways in which they interpreted questions meant to capture their motivational beliefs.

### 3.2. Materials and methods

#### 3.2.1. Measures

##### 3.2.1.1. Assessments of motivational beliefs about writing

###### 3.2.1.1.1. Binary choice survey

For the binary choice survey, testers read aloud short descriptions of two hypothetical characters with different motivational beliefs about writing, after which participants decided who they were most like. One character embodied more positive orientations to motivational beliefs, and the other character embodied more negative orientations. Participants were given the option to say that they were like neither character. At the end, testers rated participants’ overall level of understanding (1 = Did not understand the activity, 2 = May not have understood the activity, 3 = Clearly understood the activity).

In an effort to make response choices more neutral and less cognitively taxing, the response format of the binary choice survey was adapted from previous surveys designed for young children [e.g., Motivation for Reading Scale (Baker and Scher, 2002); Motivation for Reading and Writing Profile (Mata, 2011); Literacy and Writing Motivation Survey (Schrodt, 2015; Schrodt et al., 2019)]. Presenting two characters in a narrative style allowed for a more neutral presentation of response choices compared to degrees of dis/agreement. We also believed that this format would be more accessible for young children, who are often familiar with having stories read to them.

A cartoon representation of the two characters accompanied the testers’ narration of the two characters. The cartoon characters were adapted from a previous survey (Reynolds-Keefer and Johnson, 2011). The two characters only varied by height and width, allowing them to be distinguished from one another but similar enough to ensure that the visuals would not cause response bias.

Different names were selected for the two characters in each of the items. In previous work, two names were used continuously throughout the survey, such as in the case of the Literacy and Writing Motivation Survey (Schrodt, 2015; Schrodt et al., 2019), where the name “Ziggy” was used to embody more positive motivational beliefs, and “Nash” for more negative motivational beliefs. However, we felt that using the same names across all items could cause response bias for two reasons. First, participants may catch onto who is the more desirable character. Second, participants may gravitate toward a single character to remain consistent with their response of who they are most like. We also decided to give the characters culturally relevant names, as we thought that presenting too many made-up names could be cognitively taxing. We ensured that none of the names were any of the participants’ names. In this manuscript, we refer to the character with a more positive orientation to their motivational beliefs as *Character A*, and the character with a more negative orientation as *Character B*. *Characters A* and *B* were randomly ordered for each item and were not associated with a specific cartoon.

Altogether, the binary choice survey included a total of nine items intended to reflect the same constructs as the 4-point Likert-type scale survey in Study 1. We transformed the 4-point Likert-type scale survey to fit the narrative style of the binary choice survey by adapting some items from the Literacy and Writing Motivation Survey (Schrodt, 2015; Schrodt et al., 2019). In consultation with testers from Study 1, we left out a few items from the 4-point Likert-type scale survey that had caused confusion. We further made wording adjustments and added additional items to ensure that the binary choice survey items also aligned with the semi-structured interview questions (see Supplementary Table 2). Following Schrodt et al.’s (2019) procedures, a composite score was calculated by counting how many items reflected a positive orientation to motivational beliefs. Given that there were nine items, scores ranged from 0 to 9. Items were randomly ordered, and participants were randomly assigned to one of the four forms. See Supplementary Appendix B for the full measure with administration procedures.

###### 3.2.1.1.2. Challenge preference task

The challenge preference task was adapted from the Writing Challenge Task (Schrodt et al., 2019, 2022). The Writing Challenge Task was designed to expand upon typical self-report measures by aiming to capture the complexities of motivational beliefs behaviorally. We expected such behavioral tasks to be more developmentally appropriate, as they would allow participants to engage in short, concrete tasks that are more relatable (Wilson and Trainin, 2007). Our challenge preference task followed a similar procedure to the Writing Challenge Task but was shorter given the limited time we had for our testing sessions.

In the challenge preference task, participants completed short, concrete tasks, where they were asked to draw or write certain shapes, letters, or words that increased in difficulty. After each task, participants chose whether they wanted to complete a task that was more difficult, or a task that was the same level as the one they just completed. Testers did not tell participants whether their answers were correct after each task. There were five levels of difficulty in total: (1) shapes, (2) letter sounds, (3) CVC words (consonant-vowel-consonant words, e.g., “hat”), (4) two-syllable words, and (5) multi-syllable nonsense words. At the end, the testers rated participants’ level of understanding (1 = Did not understand the activity, 2 = May not have understood the activity, 3 = Clearly understood the activity). For our analyses, we examined three variables derived from this task: the highest level completed (range: 1–5), overall challenge preference profile, and preference after correctly answering an item. See Supplementary Appendix C for the full measure with administration procedures.

###### 3.2.1.1.3. Semi-structured interview

To examine kindergarteners’ perceptions of writing and motivational beliefs, we conducted semi-structured interviews. Interviews have been used in previous studies to examine motivational beliefs in kindergarteners (e.g., Nolen, 2001; Kim and Lorsbach, 2005; Hall and Axelrod, 2014; Schrodt et al., 2019). Interviews can be helpful to understand motivational beliefs from sociocultural perspectives and leverage student voices (Hall and Axelrod, 2014). More broadly, there have been calls to move beyond quantitative methods to gain a richer understanding of motivational beliefs using qualitative methods (Kim and Lorsbach, 2005).

In the semi-structured interview, testers verbally asked seven sets of questions related to motivational beliefs about writing (e.g., “First, I want you to think about some students who know how to write really well. Why do you think they know how to write well?”). Participants verbally responded to these questions. Testers asked follow-up questions as needed but stayed closely to the interview questions. Questions were adapted from the interview measure in Schrodt (2015) and Schrodt et al. (2019) and were aligned with items in the binary choice survey (see Supplementary Table 2). Upon completion, testers rated participants’ level of understanding (1 = Did not understand the activity, 2 = May not have understood the activity, 3 = Clearly understood the activity). All interviews were audio recorded and ranged in length from 3 to 8.5 min. See Supplementary Appendix D for the full measure with administration procedures.

##### 3.2.1.2. Assessment of writing

The same writing assessment from Study 1 (WRAT-5) was used in Study 2. See Study 1 for more details.

#### 3.2.2. Study procedures

Testers worked individually with participants during the school day. Participants were randomly assigned to one of the three assessment formats. 38 participants completed the binary choice survey, 37 completed the challenge preference task (*n* = 4 invalid due to tester error), and 37 completed the semi-structured interview (*n* = 3 interview recording lost due to tester error). Due to tester error, one participant completed both the challenge preference task and semi-structured interview. Given that the questions in the challenge preference task and semi-structured interview varied greatly, we decided to analyze data from both assessments.

The writing assessment was scored, then data from the binary choice survey, challenge preference task, and writing assessment were entered and analyzed using R Statistical Software (v4.2.1; R Core Team, 2022). We used *t*-tests and chi-square tests to confirm that none of the variables of interest differed by condition (treatment/control). Participants’ understanding of the assessment formats was examined in the same way as Study 1, using tester ratings.

For the binary choice survey, we used Cronbach’s alpha as a measure of internal consistency. We studied participants’ response patterns by examining the proportion of responses across items and within items. To investigate the relation between writing skills and motivational beliefs, we fit a linear regression model to predict writing skills using the composite score of the binary choice survey (in R: writing skills ~1 + motivational beliefs). We used *t*-tests to further explore whether specific survey items predicted writing skills.

For the challenge preference task, we examined three different measures of motivational beliefs that were derived from participants’ responses: the highest level completed, challenge preference profile, and preference after correctly answering an item. Response patterns for these measures were examined in a similar manner to the binary choice survey. We explored the relations between writing skills and these measures using a linear regression model or *t*-test, depending on the measure.

For the semi-structured interview, audio recordings were transcribed by a research assistant, then coded by the first and fourth authors. For each set of interview questions, we first conducted *In Vivo* Coding (Saldaña, 2013), a coding method that uses participants’ own language as codes, rather than codes developed by researchers. We then used Pattern Coding (Saldaña, 2013) to further organize the *in vivo* codes and identify emerging themes. Throughout this process, the researchers met regularly to discuss and take notes on these themes. Overall, this inductive approach to coding allowed us to identify themes that were grounded in participants’ unique experiences, aligning well with our goal of understanding motivational beliefs from their perspectives.

Given that the interviews were short and conducted only once, the researchers took caution not to over-interpret the interview responses. Prior to coding the data, the researchers were involved with collecting the raw interview data. This experience provided valuable first-hand exposure to the data and an understanding of the broader context in which the interviews were conducted. When coding and discussing the interview responses, both researchers also drew upon their former experiences as lower elementary school teachers. This experience allowed the researchers to better comprehend the interview responses.

### 3.3. Results

#### 3.3.1. Binary choice survey

Compared to the 4-point, Likert-type scale survey, the binary choice survey appeared to be easier to understand. Based on testers’ ratings, 87% of participants (*n* = 33) clearly understood the task, 13% (*n* = 5) may not have understood the task, and 0% did not understand the task. Internal consistency of the items was low (*α* = 0.49).

**Figure 3:**
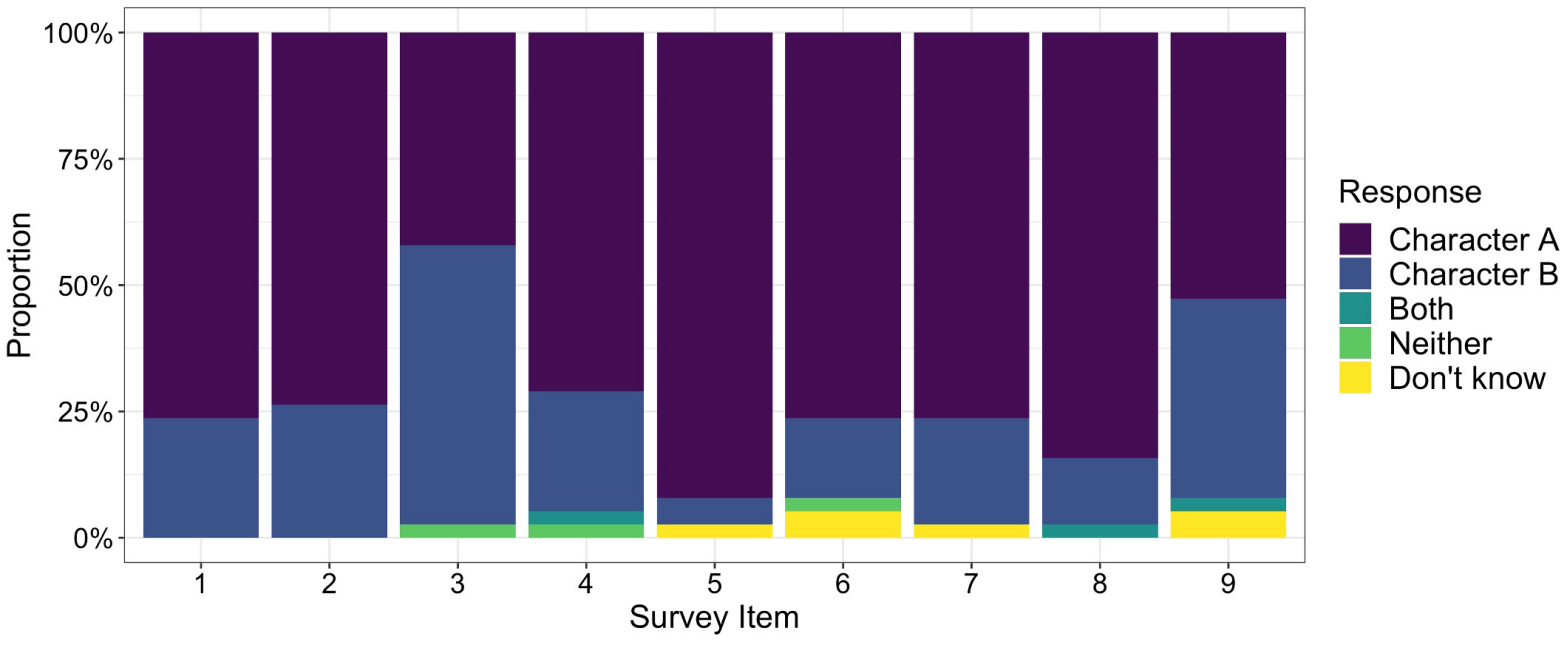
Response patterns of the 9-item, binary choice survey. Participants completed a binary choice survey by listening to narratives of two characters, then deciding which one they were most like. One of the characters embodied more positive orientations to motivational beliefs about writing (*Character A*), while the other character embodied more negative orientations (*Character B*). Some participants reported that they were like both characters, that they were like neither character, or that they did not know. Within each item, we calculated the proportion of participants who chose each response category (i.e., *Character A, Character B, Both, Neither, and Do not know*). Item numbers correspond with items included in the full measure in Supplementary Appendix B.

Despite randomizing which character type was presented first, participants tended to identify with *Character A*, which embodied more positive motivational beliefs. Across all items, participants chose *Character A* 72% of the time (*n* = 245). The responses for each of the items followed a similar pattern (see Figure 3). For seven of the nine items, over 70% of participants selected *Character A* (range: 71–92%). For the remaining two items, responses were more equally divided. For Item 3, 42% of participants resonated with *Character A* (prefers to spell challenging words), while 55% resonated with *Character B* (prefers to spell easy words). For Item 9, 53% of participants chose *Character A* (believes that their classmates who write well practiced a lot), while 39% chose *Character B* (believes that their classmates who write well have always been good at writing).

Some participants also had difficulty selecting from two binary choices (see Figure 3). Two participants responded that they resonated with both characters, and another three participants responded that they resonated with neither character. Three participants (one of whom also indicated *neither* for an item) reported that they did not know how to respond to some items.

We examined the relation between motivational beliefs and writing skills. Given the difficulty of calculating scores for participants who had answered *both*, *neither*, or *do not know* (*n* = 7), we conducted this analysis with data from the remaining 31 participants. Writing skills were not related to motivational beliefs, *F*(1, 29) = 2.80, *p* = 0.105, *r* = 0.30. We also analyzed whether writing skills could be predicted by the items with more variation in responses. After removing participants who had answered *both*, *neither*, or *do not know* (Item 3: *n* = 1, Item 9: *n* = 3), we found that there were no differences in writing skills between those who chose *Character A* and *Character B* (Item 3: *t*(35) = −1.59, *p* = 0.120; Item 9: *t*(33) = −1.33, *p* = 0.193).

#### 3.3.2. Challenge preference task

According to tester ratings, the challenge preference task also appeared to be easier for participants to understand compared to the 4-point Likert-type scale survey. Unfortunately, we lost 36% (*n* = 12) of tester ratings, given design flaws to the assessment format that made it difficult for testers to remember to rate participants’ level of understanding. Within the data that were available, 90% (*n* = 19) of participants clearly understood the task, 0% may not have understood the task, and 10% (*n* = 2) did not understand the task.

The highest level that participants completed on the challenge preference task varied: Level 1 (*n* = 10, 31%), Level 2 (*n* = 7, 21%), Level 3 (*n* = 5, 15%), Level 4 (*n* = 6, 18%), and Level 5 (*n* = 5, 15%) (see Supplementary Figure 2A). This distribution was slightly positively skewed (skewness = 0.29, *SE* = 0.41). The highest level completed was not significantly associated with writing skills, *F*(1, 31) = 3.58, *p* = 0.068, *r* = 0.32.

We also examined whether participants had a consistent pattern to their challenge preference. Specifically, we examined how many participants (1) always chose questions that were at the same level, (2) always chose questions that were more difficult, and (3) chose questions that were just right (i.e., chose questions that were at the same level after completing tasks incorrectly and chose more difficult questions after completing tasks correctly). We found that 55% (*n* = 18) fit one of these profiles, with 10 participants always choosing same-level questions, four participants always choosing difficult questions, and four participants choosing just-right questions. However, 45% of participants (*n* = 15) had inconsistent, mixed preferences, making it difficult to conduct further analyses relating challenge preference profiles to writing skills (see Supplementary Figure 2B).

Given that all participants correctly answered the first question (i.e., “Draw a square.”), we leveraged this opportunity to explore whether participants’ challenge preference after correctly answering a question was related to their writing skills. 58% of participants (*n* = 19) subsequently chose a question that was at the same level, and 42% (*n* = 14) chose a more difficult question. We found no difference in writing skills between participants of these two groups, *t*(31) = −1.12, *p* = 0.270.

#### 3.3.3. Semi-structured interview

The semi-structured interview appeared to be easier to understand than the 4-point Likert-type scale survey. Based on tester ratings, 74% of participants (*n* = 25) understood the task, 26% (*n* = 9) may not have understood the task, and 0% did not understand the task. Overall, the interview highlighted nuances that provided more context to participants’ motivational beliefs. In this section, we describe the themes that we identified from the interview responses.

##### 3.3.3.1. Positive orientations to motivational beliefs about writing

When motivational beliefs were referenced in participants’ responses, they tended to reflect positive orientations to these beliefs. In particular, participants often referenced the importance of learning and practicing (e.g., “Once you make a mistake, you, you learn the next time.”, “They [students who know how to write well] practice a lot, and they are really good now.”). Many of these responses alluded to the understanding that dosage also matters: that they need to practice and learn a lot (e.g., “I’ve been practicing a *very* long time.”, “I maybe practice at home a lot … I think I write 32 words every day.”, “I’ve been practicing and practicing and practicing, and I, and I never gave up.”). Questions also elicited responses related to self-efficacy (e.g., “I feel like I can do this. I say that to myself, and I feel like that.”), writing enjoyment (e.g., “I super love to write.”), positive self-perceptions (e.g., “I’m really good at writing words.”, “I sound it out really good.”), and persistence and hard work (e.g., “I just think of another way and do it again and again and again.”). Some participants had unique ways of describing their positive beliefs. For example, one participant referenced a necklace they were wearing, noting how this “courage necklace … has a unicorn horn, and it’s a unicorn that might give me [them] power.”

Specific nuances to these positively oriented motivational beliefs surfaced through codes that often co-occurred with these beliefs. First, participants referenced learning and practicing in specific environments with specific people that guided them (e.g., “I was teached by my dad.”). One participant shared their reasons for why they believed that one of their classmates was a less experienced writer: “Their parents probably didn’t teach them um, their writing. They don’t know very well. They didn’t go to good schools. But they’re starting to write better, but they write a ‘d’ like this. Lots of mistakes.” In other words, participants described learning and practicing as more than just an internal, cognitive process; these processes were intertwined with their external, social environments.

Second, participants’ positive motivational beliefs co-occurred with specific strategies that allowed them to have more agency in their learning. In particular, sounding out words was a specific writing strategy that many participants referred to. When asking participants why they think they are good at writing hard words, or why they think they can write hard words, they referenced specific strategies to sound out words (e.g., “I can listen to the sounds.”, “I can hear the sounds and I know the ‘c-h’ and ‘s-h’ is in there.”, “I like know sometimes there might be a silent ‘k’ at the start or silent ‘e’ at the end.”). Participants also reported using specific strategies when getting stuck or making mistakes (e.g., “I just like sound it out, and then I keep writing with the sounds … I feel like I can do this. I say that to myself, and I feel like that.”, “I try sounding it out very very slowly and take my time.”). In addition to specific writing strategies, participants occasionally referenced self-regulation strategies. For example, they shared that they take a deep breath and concentrate to accomplish difficult tasks. Similarly, participants attributed concentrating and listening to the teacher as qualities that allowed them to become good writers.

##### 3.3.3.2. Negative orientations to motivational beliefs about writing

Negative orientations to motivational beliefs were harder to capture. At times, participants responded to questions in ways that were intended to capture negatively oriented beliefs; however, the details that they included in their responses made us reconsider whether these responses were capturing negatively oriented beliefs after all. We identified four themes related to this finding, which we describe in the following paragraphs.

It was difficult to determine whether participants were expressing negative beliefs about their writing abilities, or a realistic attitude toward their current abilities as an emerging writer. Some participants responded that they are not good at writing hard words and that they cannot write hard, made-up words, because these words are tricky and have too many letters. While such responses may reflect negative beliefs, some participants further elaborated on such beliefs in ways that seemed to indicate a realistic assessment of their current writing abilities (e.g., “I don’t know really really hard words like, like that one [hard, made-up word], but I, I can write like, like the words that have five or four or six letters in them.”). By stating that they are not good at writing words or that they do not feel like they can write hard words, participants may have been reflecting on what they can and cannot do currently as a kindergarten writer.

Asking participants whether they can write hard, nonsense words also introduced another layer of complexity. Among those who reported that they cannot write these words, some participants alluded to or directly addressed the importance of knowing the meaning of the word in order to encode correctly (e.g., “You’ve never heard of it [this nonsense word], and like, there’s a bunch of letters that go together, and … it’s not real, so like, you don’t know what it’ll be and how to spell it.”, “I don’t even know what it [this nonsense word] means!,” “It [this nonsense word] has too many letters, and it’s not a word.”). In other words, these participants demonstrated a conceptualization of encoding words that was dependent on knowing the meaning of these words, rather than how motivated they were to write these words.

When participants reported that they would seek help from others when getting stuck, it was challenging to discern whether this behavior represented negative beliefs, such as a lack of self-persistence, or a reasonable awareness of other support options that were available to them. Participants specifically referred to getting help from more experienced writers, such as their teachers, grown-ups, parents, and friends. One of the participants additionally referred to getting help from technology: “When I’m writing, I ask my mom what’s the spelling, or I have a *Bixby* that, that has an *Alexa* [virtual assistant] and I, I can say ‘how do you spell that word?’” It is possible that many participants viewed help from experts, whether from people or technology, as an additional resource to guide their development as emerging writers.

Finally, some participants believed that mistakes were bad for their learning, but the various reasons they provided did not seem to stem from their motivational beliefs. One of the participants shared that mistakes could make them incorrectly learn the spelling of certain words:

[Mistakes are] bad, because sometimes I keep doing it over and over and over again … I get memorized to that, and I like think it’s the correct thing, and I’m like, I know this is right. Like that happened, happens when I write *mommy* and *daddy*. I just learned that there’s two “m”s before the “y.” And I was always writing it for *daddy* “d-a-d-y” and for *mommy* “m-o-m-y,” but now I know for *daddy*, it goes “d-a-d-d-y” and *mommy*, “m-o-m-m-y.”

Participants also expressed a concern that mistakes can cause them to get stuck. Others described how mistakes can cause confusion (e.g., “You might forget where you are.”, “It [your writing] maybe not make sense.”). Such reasons were justified and challenged the assumption that these participants hold negatively oriented motivational beliefs, simply because they stated that mistakes are bad for their learning.

##### 3.3.3.3. Kindergarteners’ perceptions of writing

In addition to the findings that directly addressed the goals of the interview, there were other related findings that further provided insight on the ways participants perceived writing. While some participants referred to spelling and writing words and sentences, others used their own words to express these processes. For example, they described “getting all the letters in” to refer to correctly spelling a word, and “making the wrong letter” and “putting some of the letters wrong” to indicate misspelling a word. To refer to the act of writing a word, they used the phrase “make the word,” while rewriting a word was described as “just erase it and make a something that is, is new.” Additionally, the act of writing a sentence was expressed as “making the sentence with like nineteen letters.” Some participants also seemed to conflate letters with words, such as when they named specific letters when asked what words they like to write.

Participants’ responses to the interview questions also highlighted the many intricate layers of the writing process. Many of them stated that sounding out and spelling words were the hardest aspects of writing, but others described additional challenges, such as writing sentences, using correct punctuation, working on handwriting, concentrating, drawing pictures to accompany their writing, and dealing with fatigue in their arms from writing a lot. Similarly, participants’ characterization of hard words also ranged. While a majority of participants viewed hard words as long words with many letters, as well as those that are difficult to spell and do not follow simple letter-to-sound correspondences, there were others that expressed additional characteristics of hard words. For example, hard words were associated with long words whose meanings may be compromised if they cannot fit onto a single line:

If there’s a lot of letters … I might kind of be focused like if I run out of space, … you would have to like, like go off the line, or you would have to go on another line … if you go on another line, it would kind of break apart and you wouldn’t like, really know how to read it.

Even at this young age, participants held an understanding of the complexities of the writing process, which were intertwined with meaning making, as well as other cognitive and motor processes that went beyond simply encoding words.

Some participants also demonstrated an awareness of their classmates’ writing abilities. In some of the interview questions, participants were prompted to think about some students who know how to write well, as well as students who do not know how to write well. While we did not intend for participants to think of or share the names of specific students with the testers, some participants shared names of their classmates. Furthermore, one participant even shared that students who know how to write well “always get compliments. They’re like, really good students.” By the end of the school year, some participants had already begun to develop a perception of their classmates’ writing abilities.

### 3.4. Study 2 summary

Overall, the three assessment formats tested in Study 2 appeared more developmentally appropriate than the 4-point Likert-type scale survey that we examined in Study 1, given the testers’ ratings of whether participants understood these three tasks. The response options in the binary choice survey were designed to be more neutral and less cognitively taxing, compared to the 4-point Likert-type scale survey. These changes likely helped more participants understand the task. However, the response options may have been oversimplified, posing a challenge in fully capturing the nuances of motivational beliefs. Participants also appeared to have an easier time understanding the challenge preference task, highlighting the promise of using a task-based, behavioral assessment in measuring motivational beliefs about writing. However, about half of the participants showed an inconsistent pattern to their challenge preference. More research is needed to further investigate whether kindergarteners’ motivational beliefs can indeed be captured through their performance on this task. Motivational beliefs, as measured by the binary choice survey and the challenge preference task, were not related to writing skills. The semi-structured interview highlighted nuances that provided more context to participants’ motivational beliefs and their experiences with writing. The interview further provided an opportunity to reevaluate behaviors that have been previously assumed to reflect negative orientations to motivational beliefs. Participants’ responses served as an important reminder that their beliefs are intricately woven into their lived experiences.

## 4. Discussion

Across two studies, we iteratively tested a total of four assessment formats to explore ways to better capture kindergarteners’ motivational beliefs about writing. In Study 1, we found that a 4-point Likert-type scale survey was too difficult to complete for most participants. In Study 2, conducted about seven months later, we examined three additional assessment formats, which we designed to be more developmentally appropriate. We found that the binary choice survey, the challenge preference task, and semi-structured interview were much easier for participants to complete compared to the 4-point Likert-type scale survey. The semi-structured interview appeared to be the most appropriate approach to capturing participants’ motivational beliefs in that it provided additional opportunity to listen to their voices. What surfaced from exploring these assessment formats was the overarching theme that kindergarteners’ thoughts appear to be multifaceted, contextually grounded, and hard to quantify.

These results are in line with the broader research base exploring the developmental appropriateness of using Likert scale surveys with young children. Mellor and Moore (2014) provide an overview of this research, noting difficulties in both the task of responding on a scale and the wording of the items. Even simple scales, such as a 3-point scale, can pose difficulties for young children who gravitate toward the extreme ends of the Likert scale. This behavior is especially prevalent when children answer abstract questions (e.g., how they feel; Chambers and Johnston, 2002). Furthermore, young children often have difficulty answering negatively oriented statements on a Likert scale (e.g., answering “true” for all statements; Marsh, 1986). These difficulties are in line with our results from the 4-point Likert-type scale survey, which appeared to be too cognitively taxing for participants.

Interestingly, among participants who were able to complete the 4-point Likert-type scale survey, there were stark differences in their responses to positively and negatively oriented items. These participants were more likely to answer toward the extreme ends on positively oriented items. However, on negatively oriented items, responses were more variable. Compared to positively oriented items that may have elicited quicker, possibly shallower responses, negatively oriented items may have promoted a deeper level of reflection among these participants. Indeed, negatively oriented items have been used more frequently than positively oriented items in related fields. Dweck’s shortened, 3-item mindset survey (Dweck et al., 1995) includes items that are all negatively oriented, compared to a longer version that includes both positively and negatively oriented items (Yeager and Dweck, 2020). While negatively oriented items may provide a more sensitive measure of motivational beliefs, they are also more cognitively taxing, limiting the number of kindergarteners who are developmentally ready for these types of items.

While we hoped that the binary choice survey would be an alternative way to measure motivational beliefs, we had difficulty striking the right balance between simplicity and sensitivity. Many more participants were able to understand this task, perhaps because it was developmentally aligned with young children’s tendency to think dichotomously (Gelman and Baillargeon, 1983). However, some participants ended up responding with *both* or *neither*, suggesting that motivational beliefs are multifaceted and difficult to assign to two rather arbitrary extremes. The composite scores that we computed from the binary choice survey were also limited, given that there were only two points on the scale.

We found it difficult to accurately operationalize motivational beliefs using the challenge preference task. Following Schrodt et al.’s (2019, 2022) Writing Challenge Task, we operationalized motivational beliefs about writing as the highest level completed on the task. However, this measure may have been confounded with writing skills. For example, participants with stronger writing skills may have completed higher levels on the challenge preference task, not because they were more motivated, but because they were more experienced writers. In fact, we observed a nonsignificant but positive trend between the highest level completed and writing skills. While prior research has interpreted such positive relations as indicative of a link between motivational beliefs and writing skills (Schrodt et al., 2022), it is important to acknowledge that this positive trend may simply be due to a confounding factor.

To disentangle participants’ writing skills from their challenge preferences, we attempted to operationalize motivational beliefs about writing in different ways. One approach we used was to examine participants’ challenge preferences after answering tasks correctly and incorrectly. We hypothesized that there could be three types of participants: those who always preferred tasks at the same level, those who always preferred more challenging tasks, and those whose preferences depended on whether the previous task was completed correctly. We found that many participants exhibited inconsistent patterns. While this does not come as a surprise given that human behavior is not always consistent, it is possible that we may have observed more consistent patterns if we had provided feedback after each item or asked participants about their confidence in their answers. Without these procedures, it was unclear whether participants were aware of their correct or incorrect responses. The results of the surveys and challenge preference task altogether highlight the difficulty of quantifying, categorizing, and operationalizing kindergarteners’ motivational beliefs.

The semi-structured interview provided an additional opportunity to understand writing and motivational beliefs from kindergarteners’ perspectives. Identifying participants with negatively oriented motivational beliefs was strikingly difficult. In typical survey measures, negatively oriented beliefs are associated with certain behaviors and thoughts, such as getting help from a teacher, feeling incompetent in writing, and believing that mistakes are detrimental to learning. While some participants expressed such behaviors and thoughts, their underlying reasons consistently pushed against the narrative that they simply lacked motivation. Instead, their responses embodied the characteristics of realistic, self-aware writers, reflective of what they are *currently* capable of as emerging writers, rather than what they *permanently* think of themselves. Participants’ interview responses further highlighted confusions around nonsense words, which differed from their broader understanding of everyday writing situated within a larger sociocultural context of meaning making. Participants also described writing processes in unique, developmentally appropriate ways that differed from the language used in the surveys. These findings underscore the need to critically reexamine common practices used to capture motivational beliefs in young children and to further reflect on the possible assumptions and interpretations that are being made from them.

The interview responses further highlighted the ways in which participants’ motivational beliefs were deeply intertwined with their learning environments. Aligned with the writer(s)-within-community model (Graham, 2018), participants viewed writing as an active, engaged process that not only includes themselves but the surrounding writing community. Participants referenced learning and practicing in specific locations (e.g., home, school) with specific people (e.g., family members, teachers) who supported their development as writers. In fact, one of the participants even mentioned the use of technology to help them write, further reminding us of the importance of considering the constantly evolving, sociocultural context of learning environments in the 21st century. The interview responses also served as a reminder that positive motivational beliefs are shaped by participants’ experiences; knowing how to independently use concrete strategies, such as sounding out words and regulating emotions, seemed to play a critical role in promoting these positive motivational beliefs. In other words, these beliefs were likely fostered by environments that supported participants’ growth as independent writers. Together, these findings highlight the situated, multifaceted nature of motivational beliefs.

### 4.1. Limitations

Our results reflect motivational beliefs about writing in a specific group of kindergarteners attending public schools in Northern California. Conducting these studies in public schools allowed us to work with a racially and socioeconomically diverse group of kindergarteners. However, our findings may not generalize to those beyond our sample. Notably, we did not have representation from Black or Native American communities. Motivational beliefs and people’s writing experiences are situated within broader communities, and thus, are likely to be influenced by an array of social, cultural, political, and historical factors. Additionally, it is important to acknowledge the larger societal context of the COVID-19 pandemic during the 2021–22 school year. Many participants likely had not attended preschool and were entering a social setting such as school for the first time. Our findings may have been impacted by this context, as participants were likely not familiar with school-specific practices, such as completing assessments and using non-verbal hand signals such as a thumbs-up to show agreement. Wearing masks also made overall communication difficult. More research is needed to further understand the experiences of kindergarteners beyond our sample.

Our studies were embedded within a larger writing intervention study, with Study 1 conducted at the beginning of the school year and Study 2 conducted at the end of the school year. Although we did not identify any differences related to the intervention provided in the larger study, the embedded nature of Studies 1 and 2 may have impacted findings. A 4-point Likert-type scale survey is likely to have posed difficulties even at the end of kindergarten, given that Likert-type scales are known to be difficult to use in even higher grade levels, such as second grade (Marsh, 1986). However, it is unclear how developmentally appropriate the binary choice survey, challenge preference task, and semi-structured interview would be at the beginning of kindergarten.

Small sample sizes may have hindered our ability to find statistically significant relations between motivational beliefs and writing skills. In Study 2, we opted to limit the sample size to examine three different assessment formats. Across all analyses, we observed weakly correlated, nonsignificant relations between motivational beliefs and writing skills. Previous studies have reported weak-to-moderate, statistically significant relations (Camacho et al., 2021). Including a larger sample of kindergarteners may have provided us with additional power to detect relations between these variables.

### 4.2. Implications and future directions for research

Overall, our results highlight the importance of deepening our understanding of motivational beliefs about writing in the context of the early elementary years. As the results suggest, commonly used surveys are difficult for kindergarteners to respond to, and survey items include assumptions of what *researchers* think are determinants of motivational beliefs in young children. Behaviors and beliefs, such as getting help from a teacher and feeling incompetent in writing, hold different meanings across contexts, such as between less experienced, developing writers and more experienced, skilled writers. We must be careful of assuming that phenomena we observe in a particular group of people, such as older children, carry the same meaning in other groups, such as younger children, and furthermore, that such phenomena can be measured validly in the same way across different populations.

Our results fit into a larger body of work that has demonstrated the importance of studying motivational beliefs within local, sociocultural contexts (e.g., Nolen, 2001, 2007; Hall and Axelrod, 2014; Graham, 2018). Although motivational beliefs are formally considered a cognitive aspect of writing (Hayes, 1996), participants’ accounts of motivational beliefs were deeply rooted in their everyday experiences, so much so that it was impossible to disentangle cognitive factors from sociocultural factors. Given such findings, it is not surprising that motivational beliefs are difficult to study in a vacuum, disengaged from their sociocultural contexts, with measures that assume that beliefs can be quantified and meaningfully placed on a single, linear spectrum. As Rowe (2023) points out in her closing paragraph of a chapter on early childhood writing, researchers should “follow the lead of young writers” (p. 199). If kindergarteners are sharing transdisciplinary accounts of motivational beliefs, researchers should also integrate sociocultural and cognitive perspectives to gain a more meaningful understanding of motivational beliefs.

In particular, it will be important to consider how interviews and observations can enrich future studies with kindergarteners. In Study 2, we decided to conduct semi-structured interviews, so that there was more alignment between the interview questions and the survey items, thereby allowing for an easier comparison of different assessment formats. While we asked probing questions to better understand participants’ line of thinking, we stuck closely to the predetermined interview questions. Interestingly, none of the participants explicitly identified themselves as writers or authors, nor did they reference storytelling or sharing stories, likely because our interview questions did not prompt such responses. In the future, it may be important to conduct more child-centered, unstructured interviews. For example, artifact-based interviewing is known to be a useful way to interview young children (cf. Danby et al., 2011). Researchers working with young multilingual children should further consider linguistically responsive interviewing techniques (cf. Kwon, 2021). Additionally, future research can benefit from incorporating interviews with family members, especially in light of the importance of considering sociocultural contexts (cf. Mortier et al., 2021). Observations will also be helpful in further studying how motivational beliefs dynamically play out in places such as the classroom (cf. Nolen, 2001, 2007) and in examining whether the opinions that young children self-report reflect what they internalize on a day-to-day basis in applied settings.

In fact, we encourage qualitative methods to be considered in research with all age groups. While older children may be better able to answer on a Likert-type scale, interviews and observations would nevertheless reveal a wealth of information that is likely to provide a richer, more accurate story of their motivational beliefs as well. For example, in discussions around growth mindset, there is a popular narrative that children who are “low-achieving” often are “less motivated” and benefit from mindset interventions to improve their academic performance^1^. While there is also the understanding that children must be given a learning environment that allows them to successfully put these beliefs into action in the first place (Yeager and Dweck, 2020), this important piece of information is often overlooked. Some people in the public^2^ have noted that “BI&POC [Black, Indigenous, and People of Color] experience systemic oppression and are more likely to develop a ‘fixed mindset,’” and that “if educators teaching ‘growth mindset’ do not take young people’s environment into account, particularly, youth experiencing white supremacy, anti-Blackness, poverty, patriarchy, and ableism, then they are engaged in glorified victim blaming” (Growth Mindset section). There is sure to be a side to motivational beliefs that we have not given children a chance to tell. Through interviews and observations, we can get closer to giving children the space to tell their stories and study motivational beliefs within social, cultural, political, and historical contexts.

On a broader scale, we suggest that the field reorient its goals for studying motivational beliefs about writing. Some previous studies have explored motivational beliefs to examine its potential in predicting writing performance (e.g., Camacho et al., 2021; Schrodt et al., 2022). Additionally, studies have further examined whether interventions aimed at enhancing motivational beliefs can supplement writing instruction and improve writing skills (e.g., Schrodt et al., 2019; Camacho et al., 2023). Yet what we found through the survey measures and interview was evidence suggesting that many kindergarteners already have positive orientations to their motivational beliefs. While this result may in part be due to social desirability bias, this finding has been reported in prior research (e.g., Mata, 2011), including an ethnographic study that went beyond self-report measures to examine motivational beliefs (Nolen, 2001).

Beyond focusing on the predictive value of motivational beliefs and the effectiveness of interventions targeting these beliefs, a bigger emphasis should be placed on examining the underlying factors that shape these beliefs in the first place. In a broader body of work, literacy motivation is known to decline as children get older, with those in lower grades more motivated than those in higher grades (Gambrell and Gillis, 2007). This same phenomenon has specifically been observed in writing as well (Knudson, 1992). This overall decline of motivational beliefs over time serves as an important reminder that negatively oriented beliefs are not purely cognitive, innate beliefs. Instead, these beliefs are dynamically shaped by the surrounding environment. Nolen (2007) conducted a longitudinal, mixed-methods study examining changes to young children’s motivational beliefs from first to third grade. Such approaches to studying motivational beliefs may help uncover factors that prevent children from maintaining positively oriented beliefs as they advance through school.

Kindergarten provides an interesting window to examine how the first years of school begin to shape young children’s motivational beliefs. In the interview, some of the participants demonstrated an awareness of their classmates’ writing abilities, even going as far as sharing that “good students” are those who “always get compliments.” It is possible that this awareness gradually results in social comparisons that further influence children’s motivational beliefs (cf. Nolen, 2001; Mata, 2011). It would be interesting to explore how this awareness forms and what role teachers play in positioning certain students as “good writers.” Such findings will be helpful in improving the field’s approach to studying motivational beliefs, steering the conversation toward shortcomings in children’s learning environments and perhaps even teacher ideologies, rather than flaws in children that must directly be “fixed” via motivation-based interventions.

### 4.3. Implications for education practice

Amid increased public attention on motivational beliefs, along with limited research on young children’s motivational beliefs about writing, we offer education practitioners a word of caution. Public media has spread the overly simplified notion that teaching children to hold positive motivational beliefs improves academic performance (see footnote 1). This simplified message makes it easy to lose sight of the greater social, cultural, political, and historical barriers that prevent certain children from putting their motivational beliefs into action. Especially in the kindergarten years, we saw little evidence suggesting that young children hold negatively oriented motivational beliefs. Given these findings, practitioners may consider focusing their attention on providing learning environments that allow young children to *maintain* positive beliefs. For example, in the interview, participants shared a variety of strategies that helped them persist through challenges, from sounding out words to using technology. Providing instruction that lets children hold more agency in their writing may promote positive motivational beliefs about writing.

Practitioners must also be careful of mistakenly assuming that some of their students “lack motivation” based on what *they* think reflects behaviors of motivation. For example, we found that being frustrated at mistakes and asking for help were not necessarily indicators of “giving up,” even though the literature may suggest so. Such assumptions can be especially dangerous in the classroom, as practitioners’ misinterpretations may position specific students as “capable learners” and others as “struggling.” In fact, such positioning can also be shaped by practitioners’ ideologies around language, race, and disability (McDermott, 1993; Hikida and Martínez, 2019). Practitioners’ beliefs may therefore impact which children can hold positive motivational beliefs.

### 4.4. Conclusion

Through Studies 1 and 2, we explored a variety of both quantitative and qualitative assessment formats of motivational beliefs about writing. This mixed-methods approach allowed for a unique analysis; data from each of the assessments complemented one another to tell a more coherent story of kindergarteners’ motivational beliefs. It can be easy to lose sight of the big picture when studying motivational beliefs in a vacuum, such as through surveys and task-based, behavioral assessments. To develop a fair and more meaningful understanding of motivational beliefs that can be applied to school settings, we must not rush to quantify motivational beliefs in young children with the goal of simply considering how these beliefs may predict writing performance. We must instead expand our explorations, integrating qualitative methods to deepen our understanding of kindergarteners’ motivational beliefs in context and to further examine the aspects of their environments that shape these beliefs in the first place. Such changes to the way we study young children’s motivational beliefs about writing are likely to reveal insights that will push the field to reconsider the ways we think and talk about motivational beliefs in older children as well.

## Data availability statement

The raw data supporting the conclusions of this article will be made available by the authors, without undue reservation.

## Ethics statement

The studies involving human participants were reviewed and approved by Stanford University. Written informed consent from the participants’ legal guardian/next of kin was not required to participate in this study in accordance with the national legislation and the institutional requirements.

## Author contributions

MT and CL planned and designed the studies. SA and CP provided input on the research design. CL and LB recruited and led communication with schools. MT, LB, and BH collected data. MT entered data and conducted statistical analyses. MT and BH conducted interview analyses. MT led the initial draft of the manuscript. CL and BH wrote sections of the manuscript. All authors revised and reviewed the manuscript.

## Funding

The research reported here was supported by the Institute of Education Sciences, U.S. Department of Education, through Grant R305A140701 to Georgia State University/Stanford University and Grant R305A200397 to Southern Methodist University. The opinions expressed are those of the authors and do not represent views of the Institute or the U.S. Department of Education.

## Conflict of interest

The authors declare that the research was conducted in the absence of any commercial or financial relationships that could be construed as a potential conflict of interest.

## Publisher’s note

All claims expressed in this article are solely those of the authors and do not necessarily represent those of their affiliated organizations, or those of the publisher, the editors and the reviewers. Any product that may be evaluated in this article, or claim that may be made by its manufacturer, is not guaranteed or endorsed by the publisher.

## Footnotes

^1^Barshay, J. (2022, December 5). *PROOF POINTS: Does growth mindset matter? The debate heats up.* The Hechinger Report. https://hechingerreport.org/proof-points-does-growth-mindset-matter-the-debate-heats-up/.

^2^Class Trouble. (2020, February 1). *A guide to coded language in education vol. I & II.* https://classtrouble.club/blogs/resonance-archives/a-guide-to-coded-language-in-education-vol-i.
