# Supplementary material for: Measuring kindergarteners’ motivational beliefs about writing: a mixed-methods exploration of alternate assessment formats
Source: Front Psychol. 2023 Aug 4;14:1217085. doi: 10.3389/fpsyg.2023.1217085 (PMC10437215; doi:10.3389/fpsyg.2023.1217085)
Supplement: Supplementary file 1 [file Data_Sheet_1.pdf]

frontiers

Supplementary Material

1 Supplementary Figures and Tables

Supplementary Table 1

Comparison Between Items in the Reading Mindset Measure and 4-Point Likert-Type Scale Survey

Item	Reading Mindset Measure[1]	Four-Point Likert-Type Scale Survey
1	If a book is hard to read, I stop reading it.	If a word is hard to write, I stop writing it.
2	I feel like I am one of the worst readers in my class.	I feel like I am one of the worst writers in my class.
3	If I have to read out loud in class, I feel scared.	If I have to write a word in front of the class, I feel scared.
4	If I make a lot of mistakes while reading, I quit trying.	If I make a lot of mistakes while writing words, I quit trying.
5	When I have to work hard at reading, it makes me feel like I am not very smart.	When I have to work hard at writing words, it makes me feel like I am not very smart.
6	When someone reads better than me, I'm jealous.	When someone writes words better than me, I'm jealous.
7	I don't like when my teacher corrects me when I'm reading.	I don't like when someone corrects me when I'm writing words.
8		I think I can keep getting better at writing words.
9		I like writing words even if I make a lot of mistakes.
10		I can always become a better writer if I work hard.

[1] Al Otaiba et al., 2020; Tock et al., 2021

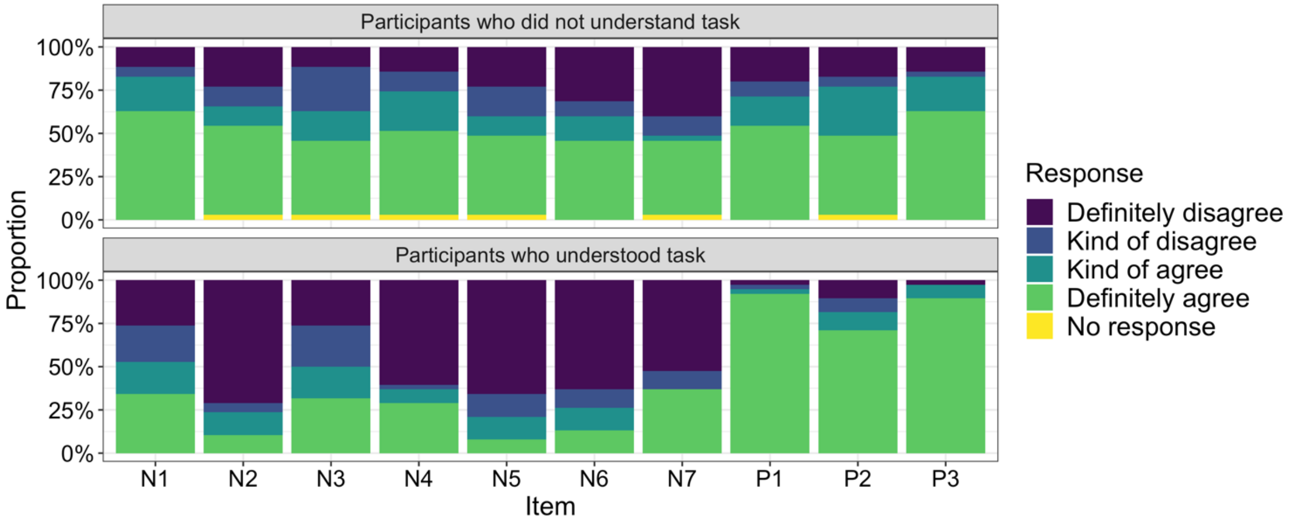

Supplementary Figure 1. Response patterns of the 10-item, 4-point Likert-type scale survey by participants' level of understanding (did not understand task/understood task). Participants responded on a 4-point Likert-type scale, after listening to testers read out survey items that were intended to measure motivational beliefs about writing. Testers rated how well participants understood the task (n = 35 did not understand task, n = 38 understood task). Within each item and level of understanding, we calculated the proportion of participants who chose each response category (i.e., *Definitely disagree*, *Kind of disagree*, *Kind of agree*, *Definitely agree*, and *No response*). The surveys included seven negatively oriented items (tagged with "N") and three positively oriented items (tagged with "P"). Survey items were as follows: N1) *If a word is hard to write, I stop writing it.*, N2) *I feel like I am one of the worst writers in my class.*, N3) *If I have to write a word in front of class, I feel scared.*, N4) *If I make a lot of mistakes while writing words, I quit trying.*, N5) *When I have to work hard at writing words, it makes me feel like I am not very smart.*, N6) *When someone writes words better than me, I'm jealous.*, N7) *I don't like when someone corrects me when I'm writing words.*, P1) *I think I can keep getting better at writing words.*, P2) *I like writing words even if I make a lot of mistakes.*, and P3) *I can always become a better writer if I work hard.*

Supplementary Table 2

Comparison Between the Binary Choice Survey Items and the Semi-Structured Interview Questions

Binary Choice Survey	Semi-Structured Interview
[A] and [B] have classmates who can write really well. [A] thinks they can write well, because they practiced a lot. [B] thinks they can write well, because they've always been good at writing. Who are you most like?	First, I want you to think about some students who know how to write really well. Why do you think they know how to write well?<br><br>Now think about some students who *don't* know how to write very well. Why do you think they *don't* know how to write well?
[A] thinks they are good at writing hard words. [B] thinks they are not good at writing hard words. Who are you most like?	Are you good at writing hard words? What makes you feel this way?
[B] prefers to spell easy words. [A] prefers to spell challenging words. Who are you most like?	What kind of words do you like to write?
[A] and [B] are spelling a hard word like *chi-mu-rie-noo-shalt*. [A] thinks they can get most of the letters right. [B] thinks they can't get any letters right. Who are you most like?	Let's say you're writing a really hard, made-up word like *chi-mu-rie-noo-shalt*. Do you think you can get most of the letters right? Why?
When [B] can't spell a word, they don't feel good about themselves. When [A] can't spell a word, they feel okay. Who are you most like?<br><br>When [B] can't spell a word, they have a hard time believing that they will get better at spelling. When [A] can't spell a word, they still think that they'll get better at spelling. Who are you most like?	What is the hardest thing about writing? How do you feel when you get stuck while you're writing?
[A] and [B] don't know how to spell a word on their own. [A] still tries to spell the word anyways. [B] stops trying and asks someone for help. Who are you most like?	What do you do when you get stuck?
[B] is afraid of making mistakes when they write. [A] is okay with making mistakes when they write. Who are you most like?	What are some mistakes you make in writing? How do you feel when you make a mistake? What do you do when you make a mistake?

Supplementary Table 2 (continued)

Binary Choice Survey	Semi-Structured Interview
[B] thinks mistakes are bad for their learning. [A] thinks mistakes are good for their learning. Who are you most like?	Do you think mistakes are good or bad for your learning?
	If you were going to tell a younger student how to write, what would you tell them?

Note. Survey items and interview questions were adapted from Schrodt (2015) and Schrodt et al. (2019).

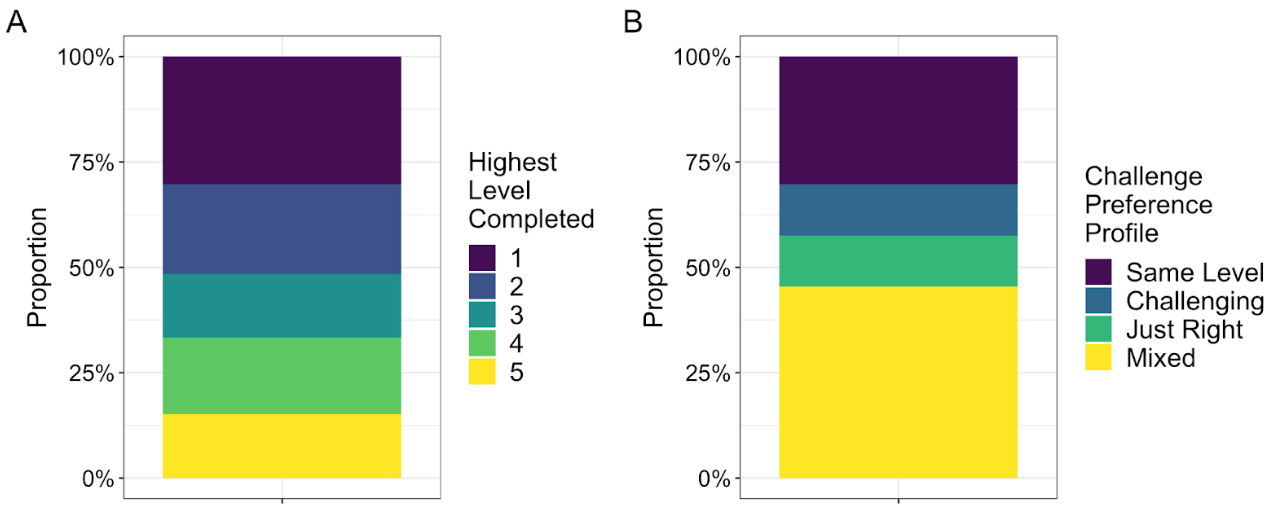

Supplementary Figure 2. Response patterns of the challenge preference task. Participants completed short writing tasks that increased in difficulty. After each task, participants chose whether to do another task that was more challenging or the same level as the one they just completed. The response patterns show the proportion of participants by A) the highest level that they completed (i.e., Levels 1–5), and B) their challenge preference profile. *Same Level* includes participants who always preferred questions at the same level. *Challenging* includes participants who always preferred challenging questions. *Just Right* includes participants who chose questions that were at the same level after completing tasks incorrectly and chose more difficult questions after completing tasks correctly. *Mixed* includes participants who had an inconsistent challenge preference.

2 Supplementary Appendices

Supplementary Appendix A

Four-Point Likert-Type Scale Survey

Tester Form

Preparation:
Place the survey in front of the student and give them a pencil.

Answer Choice Explanation:
For this activity, you're going to circle how much you disagree or agree out of these four responses.

- [point to definitely disagree] **This thumb pointing all the way down means "*definitely* disagree." That means you disagree *a lot*.**
- [point to kind of disagree] **This thumb that is sort of pointing down means "*kind of* disagree." That means you disagree *a little*.**
- [point to kind of agree] **This thumb that is sort of pointing up means "*kind of* agree." That means you agree *a little*.**
- [point to definitely agree] **And this thumb that is pointing all the way up means "*definitely* agree." That means you agree *a lot*.**

Practice Question:
Let's start with a practice. "I like dogs." Do you definitely disagree, kind of disagree, kind of agree, or definitely agree? Choose the response that best fits what you think.

Survey Items:
Ready for some more? There is no right or wrong answer, so don't be afraid to choose what you really think! Listen carefully, then take your time to choose the response that best fits what you think. Here we go!

[point to 1] **If a word is hard to write, I stop writing it. Do you *definitely* disagree, *kind of* disagree, *kind of* agree, or *definitely* agree?**
[point to 2] **I feel like I am one of the worst writers in my class.**
[point to 3] **If I have to write a word in front of the class, I feel scared.**
[point to 4] **If I make a lot of mistakes while writing words, I quit trying.**
[point to 5] **When I have to work hard at writing words, it makes me feel like I am not very smart.**
[point to 6] **I think I can keep getting better at writing words.**
[point to 7] **I like writing words even if I make a lot of mistakes.**
[point to 8] **When someone writes words better than me, I'm jealous.**
[point to 9] **I don't like when someone corrects me when I'm writing words.**
[point to 10] **I can always become a better writer if I work hard.**

Note. Most items were adapted from the Reading Mindset Measure (Al Otaiba et al., 2020; Tock et al., 2021).

Great job! We are all done!

Tester Rating:

Circle the student's level of understanding: 1 2 3
1 - Did not understand the activity.
2 - May not have understood the activity.
3 - Clearly understood the activity.

Other Notes as needed:

Student Form

Student Number/Name: ___________________

Circle how much you disagree or agree out of these four responses.

PRACTICE: I like dogs.

Definitely DISAGREE!	Kind of Disagree	Kind of Agree	Definitely AGREE!

---

1. If a word is hard to write, I stop writing it.

Definitely DISAGREE!	Kind of Disagree	Kind of Agree	Definitely AGREE!

2. I feel like I am one of the worst writers in my class.

Definitely DISAGREE!	Kind of Disagree	Kind of Agree	Definitely AGREE!

3. If I have to write a word in front of the class, I feel scared.

Definitely DISAGREE!	Kind of Disagree	Kind of Agree	Definitely AGREE!

4. If I make a lot of mistakes while writing words, I quit trying.

Definitely DISAGREE!	Kind of Disagree	Kind of Agree	Definitely AGREE!

5. When I have to work hard at writing words, it makes me feel like I am not very smart.

Definitely DISAGREE!	Kind of Disagree	Kind of Agree	Definitely AGREE!

6. I think I can keep getting better at writing words.

Definitely DISAGREE!	Kind of Disagree	Kind of Agree	Definitely AGREE!

7. I like writing words even if I make a lot of mistakes.

Definitely DISAGREE!	Kind of Disagree	Kind of Agree	Definitely AGREE!

8. When someone writes words better than me, I'm jealous.

Definitely DISAGREE!	Kind of Disagree	Kind of Agree	Definitely AGREE!

9. I don't like when someone corrects me when I'm writing words.

Definitely DISAGREE!	Kind of Disagree	Kind of Agree	Definitely AGREE!

10. I can always become a better writer if I work hard.

Definitely DISAGREE!	Kind of Disagree	Kind of Agree	Definitely AGREE!

Supplementary Appendix B

Binary Choice Survey

Tester Form

Student Number/Name: _________________

Materials:

- Students need visuals of the characters to point to. (An example can be obtained from the first author.)
- Tester needs this form and a pencil.

Directions:

- Instructions: **For this activity, I'm going to share what some students think, and you can tell me who you are most like. You can either tell me their names or point to one of them.** [point to first two students] **If you don't think you're like either student, just let me know. Also let me know if you want me to repeat what I said. Finally, there's no right or wrong answer, so you're welcome to let me know what you think.**
- When students don't think they are like either student, or tell you they don't know:
    - If you have time, ask why they can't decide and add their response in the notes.

	Items (N = Neither, DK = Don't Know)	Other Observations and Notes
1	[B] thinks mistakes are bad for their learning. [A] thinks mistakes are good for their learning. Who are you most like?<br><br>[B] / [A] / N / DK	
2	[A] thinks they are good at writing hard words. [B] thinks they are not good at writing hard words. Who are you most like?<br><br>[A] / [B] / N / DK	
3	[B] prefers to spell easy words. [A] prefers to spell challenging words. Who are you most like?<br><br>[B] / [A] / N / DK	
4	[A] and [B] don't know how to spell a word on their own. [A] still tries to spell the word anyways. [B] stops trying and asks someone for help. Who are you most like?<br><br>[A] / [B] / N / DK	

5	[B] is afraid of making mistakes when they write. [A] is okay with making mistakes when they write. Who are you most like?<br><br>[B] / [A] / N / DK	
6	When [B] can't spell a word, they don't feel good about themselves. When [A] can't spell a word, they feel okay. Who are you most like?<br><br>[B] / [A] / N / DK	
7	When [B] can't spell a word, they have a hard time believing that they will get better at spelling. When [A] can't spell a word, they still think that they'll get better at spelling. Who are you most like?<br><br>[B] / [A] / N / DK	
8	[A] and [B] are spelling a hard word like *chi-mu-rie-noo-shalt.* [A] thinks they can get most of the letters right. [B] thinks they can't get any letters right. Who are you most like?<br><br>[A] / [B] / N / DK	
9	[A] and [B] have classmates who can write really well. [A] thinks they can write well, because they practiced a lot. [B] thinks they can write well, because they've always been good at writing. Who are you most like?<br><br>[A] / [B] / N / DK	

Note. The response format was adapted from Baker & Scher (2002), Mata (2011), Schrodt (2015), and Schrodt et al. (2019). Items were adapted from Schrodt (2015) and Schrodt et al. (2019).

Tester Rating:

Circle the student's level of understanding: 1 2 3
1 - Did not understand the activity.
2 - May not have understood the activity.
3 - Clearly understood the activity.

Other Notes as needed:

Supplementary Appendix C

Challenge Preference Task

Tester Form

Student Number/Name: ________________

Overview: Students will be asked to complete writing tasks that are either at the same level or a more difficult level. After each shape/letter/word they write, students will have the option to choose something harder or just the same. The chart below has arrows (→ ↓) to guide you through the prompts. Stop administering when the arrows lead you to the STOP zone. Students will write items in the boxes on the student form.

Materials:

- Child needs student form and pencil.
- Tester needs this paper and pencil.

Directions:

- **For this activity, you'll be writing some things, letters or words. First, can you draw a square here?** [point to first box on worksheet]
- During each task:
  - Fill out the cell in the chart. Do not tell them if they got a word correct or incorrect.
  - If a student asks for help, say: **That's okay - just do your best!**
- After each task: **Would you like something harder, or just the same?**
  - Follow arrows in the chart, depending on their response
    - If they choose *harder*, move directly down to next level.
    - If they choose *just the same*, move directly across (to the right).
    - Easier is not an option, but if they say easier, move directly across (don't go to an easier level)
  - Stop administering once the arrows lead you to the STOP zone.

Note. This task was adapted from the Writing Challenge Task (Schrodt et al., 2019, 2022).

***** **Flip page for chart.** *****

	Task 1	Task 2	Task 3	
**Level 1: Shapes**	Draw a square.<br>**Is it correct?** (circle one)<br>Yes / No / Questionable<br>**Notes:**<br><br>Would you like something harder, or just the same?<br>(circle one & follow arrow)<br>*easy* →<br>*same* →<br>*challenging* ↓	Draw a triangle.<br>**Is it correct?** (circle one)<br>Yes / No / Questionable<br>**Notes:**<br><br>Would you like something harder, or just the same?<br>(circle one & follow arrow)<br>*easy* →<br>*same* →<br>*challenging* ↓	Draw a circle.<br>**Is it correct?** (circle one)<br>Yes / No / Questionable<br>**Notes:**<br><br>Would you like something harder, or just the same?<br>(circle one & follow arrow)<br>*easy* →<br>*same* →<br>*challenging* ↓	**STOP**
**Level 2: Letter Sounds**	Write the letter that says /g/<br>**Is it correct?** (circle one)<br>Yes / No / Questionable<br>**Notes:**<br><br>Would you like something harder, or just the same?<br>(circle one & follow arrow)<br>*easy* →<br>*same* →<br>*challenging* ↓	Write the letter that says /p/<br>**Is it correct?** (circle one)<br>Yes / No / Questionable<br>**Notes:**<br><br>Would you like something harder, or just the same?<br>(circle one & follow arrow)<br>*easy* →<br>*same* →<br>*challenging* ↓	Write the letter that says /s/<br>**Is it correct?** (circle one)<br>Yes / No / Questionable<br>**Notes:**<br><br>Would you like something harder, or just the same?<br>(circle one & follow arrow)<br>*easy* →<br>*same* →<br>*challenging* ↓	**STOP**
**Level 3: CVC Words**	Write the word: bed.<br>**Is it correct?** (circle one)<br>Yes / No / Questionable<br>**Notes:**<br><br>Would you like something harder, or just the same?<br>(circle one & follow arrow)<br>*easy* →<br>*same* →<br>*challenging* ↓	Write the word: hat.<br>**Is it correct?** (circle one)<br>Yes / No / Questionable<br>**Notes:**<br><br>Would you like something harder, or just the same?<br>(circle one & follow arrow)<br>*easy* →<br>*same* →<br>*challenging* ↓	Write the word: dog.<br>**Is it correct?** (circle one)<br>Yes / No / Questionable<br>**Notes:**<br><br>Would you like something harder, or just the same?<br>(circle one & follow arrow)<br>*easy* →<br>*same* →<br>*challenging* ↓	**STOP**

**Level 4: Two-Syllable Words**	Write the word: shower.<br>**Is it correct?** (circle one)<br>Yes / No / Questionable<br>**Notes:**<br><br>Would you like something harder, or just the same?<br>(circle one & follow arrow)<br>*easy →*<br>*same →*<br>*challenging ↓*	Write the word: sandwich.<br>**Is it correct?** (circle one)<br>Yes / No / Questionable<br>**Notes:**<br><br>Would you like something harder, or just the same?<br>(circle one & follow arrow)<br>*easy →*<br>*same →*<br>*challenging ↓*	Write the word: birthday.<br>**Is it correct?** (circle one)<br>Yes / No / Questionable<br>**Notes:**<br><br>Would you like something harder, or just the same?<br>(circle one & follow arrow)<br>*easy →*<br>*same →*<br>*challenging ↓*	**S<br>T<br>O<br>P**
**Level 5: Multi-Syllable Non-sense Words**	Write the made-up word: chi-mu-rie-noo-shalt.<br>**Is it correct?** (circle one)<br>Yes / No / Questionable<br>**Notes:**<br><br>Would you like something harder, or just the same?<br>(circle one & follow arrow)<br>*easy →*<br>*same →*<br>*challenging ↓*	Write the made-up word: sha-so-kee-geh-maj.<br>**Is it correct?** (circle one)<br>Yes / No / Questionable<br>**Notes:**<br><br>Would you like something harder, or just the same?<br>(circle one & follow arrow)<br>*easy →*<br>*same →*<br>*challenging ↓*	Write the made-up word: ther-ba-zie-ner-say.<br>**Is it correct?** (circle one)<br>Yes / No / Questionable<br>**Notes:**<br><br>Would you like something harder, or just the same?<br>(circle one & follow arrow)<br>*easy →*<br>*same →*<br>*challenging ↓*	**S<br>T<br>O<br>P**
		**STOP**		

Tester Rating:

Circle the student's level of understanding: 1 2 3
1 - Did not understand the activity.
2 - May not have understood the activity.
3 - Clearly understood the activity.

Other Notes as needed:

Student Form

Student Number/Name: ______________________

Supplementary Appendix D

Semi-Structured Interview

Tester Form

Student Number/Name: _________________

Materials:

- Students don't need materials.
- Tester needs a voice recorder, this paper, and a pencil.

Instructions:

- **For this activity, I'll ask some questions about writing. There's no right or wrong answer, so you are welcome to share what you really think. I'm going to record this, so I can go back and listen to what you said. Is that okay with you?**
- Start Recording
    - On/Off button is on the left side of the recorder. Slide it down to turn on.
    - Should already be in *interview mode*. If not, Option > Scene Select > Interview
    - Press the red REC button.
        - If you need to pause, press the same REC button again, and press again to resume.
        - Once complete, save by pressing the STOP button.
    - Say: **Your Name, Student Name, School Name, Teacher Name, Date**

******* Flip page for interview questions. *******

Interview Questions:

1. First, I want you to think about some students who know how to write really well. Why do you think they know how to write well?

2. Now think about some students who *don't* know how to write very well. Why do you think they *don't* know how to write well?

3. Are you good at writing hard words? Why? What kind of words do you like to write?

4. Let's say you're writing a really hard, made-up word like *chi-mu-rie-noo-shalt*. Do you think you can get most of the letters right? Why?

5. What is the hardest thing about writing? How do you feel when you get stuck while you're writing? What do you do when you get stuck?

6. What are some mistakes you make in writing? How do you feel when you make a mistake? What do you do when you make a mistake? Do you think mistakes are good or bad for your learning? Why?

7. If you were going to tell a younger student how to write, what would you tell them?

Note. Interview questions were adapted from Schrodt (2015) and Schrodt et al. (2019).

Tester Rating:

Circle the student's level of understanding: 1 2 3
1 - Did not understand the activity.
2 - May not have understood the activity.
3 - Clearly understood the activity.

Other Notes as needed:
